# The ruxolitinib effect: understanding how molecular pathogenesis and epigenetic dysregulation impact therapeutic efficacy in myeloproliferative neoplasms

**DOI:** 10.1186/s12967-018-1729-7

**Published:** 2018-12-17

**Authors:** Graeme Greenfield, Suzanne McPherson, Ken Mills, Mary Frances McMullin

**Affiliations:** 1 Centre for Cancer Research and Cell Biology, Queen’s University Belfast, Belfast, UK; 2 Centre for Medical Education, Queen’s University Belfast, Belfast, UK

**Keywords:** Myeloproliferative neoplasms, Polycythaemia vera, Essential thrombocythemia, Primary myelofibrosis, Ruxolitinib, Epigenetics

## Abstract

The myeloproliferative neoplasms (MPN), polycythaemia vera (PV), essential thrombocythemia (ET) and primary myelofibrosis (PMF) are linked by a propensity to thrombosis formation and a risk of leukaemic transformation. Activation of cytokine independent signalling through the JAK/STAT cascade is a feature of these disorders. A point mutation in exon 14 of the *JAK2* gene resulting in the formation of the *JAK2* V617F transcript occurs in 95% of PV patients and around 50% of ET and PMF patients driving constitutive activation of the JAK/STAT pathway. Mutations in *CALR* or *MPL* are present as driving mutations in the majority of remaining ET and PMF patients. Ruxolitinib is a tyrosine kinase inhibitor which inhibits JAK1 and JAK2. It is approved for use in intermediate and high risk PMF, and in PV patients who are resistant or intolerant to hydroxycarbamide. In randomised controlled trials it has demonstrated efficacy in spleen volume reduction and symptom burden reduction with a moderate improvement in overall survival in PMF. In PV, there is demonstrated benefit in haematocrit control and spleen volume. Despite these benefits, there is limited impact to induce complete haematological remission with normalisation of blood counts, reduce the mutant allele burden or reverse bone marrow fibrosis. Clonal evolution has been observed on ruxolitinib therapy and transformation to acute leukaemia can still occur. This review will concentrate on understanding the clinical and molecular effects of ruxolitinib in MPN. We will focus on understanding the limitations of JAK inhibition and the challenges to improving therapeutic efficacy in these disorders. We will explore the demonstrated benefits and disadvantages of ruxolitinib in the clinic, the role of genomic and clonal variability in pathogenesis and response to JAK inhibition, epigenetic changes which impact on response to therapy, the role of DNA damage and the role of inflammation in these disorders. Finally, we will summarise the future prospects for improving therapy in MPN in the JAK inhibition era.

## Background

Myeloproliferative diseases were first described by Dameshek in the 1950’s by linking high blood counts and disease phenotypes [1]. Since then, the World Health Organisation (WHO) has defined polycythaemia vera (PV), essential thrombocythemia (ET), primary myelofibrosis (PMF) and pre-fibrotic PMF as the most common Philadelphia chromosome negative myeloproliferative neoplasms (MPN) [2]. The polyclonal proliferation of myeloid cells in normal health is replaced by an abnormal monoclonal proliferation resulting in an overproduction of red blood cells in PV, platelets in ET and bone marrow fibrosis in PMF. There are common phenotypic characteristics, with a predisposition to thromboembolic and haemorrhagic pathologies and a risk of progression to myelofibrosis (MF) or leukaemic/blast phase transformation.

A point mutation in exon 14 of the *JAK2* gene resulting in the formation of the *JAK2* V617F transcript and conformational shift of the resulting JH2 pseudo-kinase domain of JAK2 drives constitutive activation of the JAK/STAT pathway. This is identified in approximately 95% of PV cases and around 50% of ET and PMF cases [3, 4]. The remaining 5% of PV patients are almost entirely accounted for by mutations in exon 12 of the *JAK2* gene. The majority of remaining ET and PMF cases have JAK/STAT activation resulting from driving mutations in *CALR* or *MPL* genes [5–7]. A small number of ET and PMF cases are “triple negative” [8].

The introduction of targeted JAK inhibition (JAKi) within the last decade has brought an element of precision medicine and an attempt at disease modification to the MPN field. Ruxolitinib (RUX) is a JAK1/JAK2 inhibitor which has been approved by the US Food and Drug Agency and European Medicines Agency for the treatment of intermediate and high risk MF and second line for PV patients resistant or intolerant to hydroxycarbamide (HU).

This review will concentrate on understanding the molecular aspects and epigenetic dysregulation impacting on the clinical effects of RUX in MPN. Understanding the limitations of JAKi at a genomic and cellular level highlight the challenges to improving therapeutic options in MPN. We will explore the demonstrated benefits and disadvantages of RUX in the clinic and the role that genomic changes, clonal variability and epigenetics have in pathogenesis of MPN and response to JAKi. We will also consider how JAKi interacts with the role of DNA damage and inflammation in these disorders. Improving therapy in MPN in the JAKi era is an unmet need and we will summarise future prospects.

## Main text

### JAK inhibition in the clinic

RUX has demonstrated efficacy in spleen volume reduction and symptom burden reduction when compared against best available therapy (BAT) or placebo in intermediate or high risk MF [9–14]. There is a rapid recurrence of symptoms evident in MF patients on disease interruption [9]. Improved overall survival (OS) was also observed in the initial phase 3 studies. A combined analysis of the COMFORT-I AND COMFORT-II studies demonstrated a 30% risk reduction of death and a significant survival advantage in those originally randomised to RUX in comparison to those crossing over [15]. However, the nature of early cross-over from BAT or placebo to RUX in the control arm and insufficient power to assess the survival benefit mean that the impact on OS has been questioned by some [16, 17]. In PV, improved haematocrit control and spleen volume reduction have been demonstrated in comparison to best available therapy [18–21]. The only randomised control trial undertaken comparing RUX to best available therapy in ET did not show any benefit as second line therapy in patients intolerant or resistant to HU [22]. An earlier phase 2 study of RUX in ET did suggest an improvement in symptom burden in the same second line setting, but did not include a control arm [23]. Table 1 summarises the findings of the key clinical trials undertaken to date.

**Table 1:** Randomised control trials of ruxolitinib in MPN

TRIAL	MPN	Trial	Endpoints	Long term outcomes	Significant toxicities	Genomic effects 1—driver mutations	Genomic effects 2—additional mutations	References
COMFORT 1	PMF Post ET-MF Post PV-MF Intermediate 2 or High Risk	Ruxolitinib (n = 155) v Placebo (n = 154)	SVR ≥ 35% 24 weeks RUX = 41.9% Placebo = 0.7% TSS reduction ≥ 50% at 24 weeks RUX = 45.9% Placebo = 5.3%	Median OS RUX = NR Placebo = 200 weeks Spleen response duration RUX = 168.3 weeks	Grade ¾ Anaemia RUX = 45.2% Placebo = 19.2% Thrombocytopenia RUX = 12.9% Placebo = 1.3% Neutropenia RUX = 7.1% Placebo = 2.0%	*JAK2* V617F positive RUX = 73% Placebo = 80% No difference between *JAK2* V617F mutation positive or negative patients	Not available	[9–11]
COMFORT 2	PMF Post ET-MF Post PV-MF Intermediate 2 or High Risk	Ruxolitinib (n = 146) v BAT (n = 73)	SVR ≥ 35% 48 weeks RUX = 28% BAT = 0%	Median OS RUX = NR BAT = 4.1 years Spleen response duration RUX = 3.2 years	Grade ¾ Anaemia RUX = 46.1% Thrombocytopenia RUX = 18.8% Neutropenia RUX = 8.9% Lymphopenia RUX = 31.4%	110/146 RUX. pts *JAK2* V617F positive Median allele burden = 84% Allelle burden reduction ≥ 20% = 38.3% weeks 168	High molecular risk v low molecular risk (defined previously in [25]) SVR ≥ 35% 48 weeks HMR = 26.1% LMR = 35.0% Mean SVR 48 weeks HMR = − 23.5% LMR = − 30.6% No mutation individually correlated KM estimate Survival 144 weeks HMR RUX = 0.79 HMR BAT = 0.58 LMR RUX = 0.85 LMR BAT = 0.71 HMR pts have HR 0.57 (CI 0.30–1.08) of death No data on *CALR*	[13, 14]
JUMP	PMF Post ET-MF Post PV-MF Intermediate 1, intermediate 2 or high risk	Single arm Ruxolitinib study (n = 1144)	SLR ≥ 50% 48 weeks RUX = 62.3% Spleen response duration RUX = NR	OS probability at 48 weeks RUX = 94% PFS probability at 48 weeks RUX = 91%	Grade ¾ Anameia = 33% Thrombocytopenia = 12.5% Neutropenia = 3.9%	Not available	Not available	[12]
RESPONSE 1	PV HU intolerant/resistant with splenomegaly	Ruxolitinib (n = 110) v BAT (n = 112)	Haematocrit control and SVR ≥ 35% 32 weeks RUX = 20.9% BAT = 0.9% Haematocrit Control 32 weeks RUX = 60.0% BAT = 19.6% SVR ≥ 35% 32 weeks RUX = 38.2% BAT = 0.9%	Thromboembolic rate RUX = 1.8/100pt years BAT = 8.2/100pt years CHR at 32 weeks RUX = 23.6% BAT = 8.9% Maintained CHR at 80 weeks RUX = 69%	Grade 3/4 80 weeks Anaemia RUX = 0.9% Thrombocytopenia RUX = 2.6% Neutropenia RUX = 0.4% Lymphopenia RUX = 9.7%	*JAK2* V617F allele burden 32 weeks RUX = − 12.2% BAT = + 1.2% *JAK2* V617F allele burden 80 weeks RUX = − 22.0% *JAK2* V617F allele burden 208 weeks max reduction RUX = − 35.9% Crossover = − 21.2%	CMR/PMR possible in patients with *ASXL1*, *TET2*, *JAK3*, *SOCS1* mutations	[20, 21, 26]
RESPONSE 2	PV HU intolerant/resistant without splenomegaly	Ruxolitinib (n = 74) v BAT (n = 75)	Haematocrit control 28 weeks RUX = 62% BAT = 19%	Maintenance haematocrit response RUX = 78% CHR Maintained at 80 weeks RUX = 24.3% BAT = 2.7% TSS reduction ≥ 50% 80 weeks RUX = 45%	Grade 3/4 80 weeks Anaemia RUX = 0% Thrombocytopenia RUX = 0% Hypertension RUX = 6.8% BAT = 5.7%	*JAK2* V617F Burden 28 weeks RUX = − 4.7% BAT = − 2.0% *JAK2* V617F Burden 80 weeks RUX = − 9.7%	Not available	[18, 19]
MAJIC ET	ET HU intolerant/resistant	Ruxolitinib (n = 58) v BAT (n = 52)	Complete response within 1 year RUX = 46.6% BAT = 44.2%	Thromboembolic events in 2 years RUX = 17.2% BAT = 5.8%		*JAK2* V617F Burden No change in mean allele burden in either treatment arm	Not available	[22]

*CHR* complete haematological remission, *CMR* complete molecular response, *HMR* high molecular risk, *KM* Kaplan Meier, *NR* not reached, *LMR* low molecular risk, *OS* overall survival, *PFS* progression free survival, *PMR* partial molecular response,* SLR* spleen length response,*SVR* spleen volume response,* TSS* total symptom score

The efficacy of RUX is variable across the MPN phenotype with clear benefits for selected patients. Despite the direct targeting of the JAK/STAT signalling it is limited as a true disease modifying therapy. As demonstrated in Table 1 reductions in mutant allele burden are minimal to moderate yet are often sustained on therapy. Transformations to both secondary MF and acute leukaemia have both been observed on ruxolitinib therapy with no studies with adequate follow-up or statistical power to determine if there is any deviation in frequency from this aspect of disease course. There is a suggestion of lower rates of thrombosis in the RESPONSE trials although statistical power is again an issue [20]. In MF, sustained RUX therapy appears to improve the odds of stabilisation of bone marrow fibrosis and reduce the chance of worsening fibrosis in a number of patients but does not bring about any significant reversal [24]. Allogeneic haematopoietic stem cell transplantation offers the only possibility of disease modification and cure. However, the majority of patients will not be considered suitable for this due to the associated toxicities.

### Genomic impacts on pathogenesis and JAK inhibition efficacy

Constitutive activation of the JAK/STAT signalling pathway is key to the development of the MPN phenotype in all mutant backgrounds. Regardless of clinical phenotype or somatic mutation status, all MPN patients show a characteristic gene expression signature resulting from JAK/STAT activation [8]. *JAK2* V617F mutations can drive a spectrum of disease across the PV, ET or PMF phenotypes through activation of erythropoietin receptor (EPOR), thrombopoietin receptor (MPL) and granulocyte-colony stimulating factor receptor (G-CSFR) receptors present on differing stages of a maturing myeloid cell. *JAK2* exon 12 mutations drive a predominant erythrocytosis possibly through predominant activation of EPOR signalling pathways. Clonal dominance of homozygosity or heterozygosity of *JAK2* V617F, the presence and order of acquisition of co-operating mutations and additional factors such as iron deficiency and gender can impact on the resulting phenotype [25]. *CALR* and *MPL* mutations result in an ET or PMF phenotype through activation of the MPL receptor [26]. All drivers appear to be largely mutually exclusive of each other although bi-clonal disease can occur [27]. *JAK2*, *CALR*, *MPL* mutations are sufficient in themselves to produce an MPN phenotype in murine models although these are often polyclonal in nature thus not entirely representative of a true MPN [28]. *JAK2* V617F and *CALR* mutations are detectable in the long term haematopoietic stem cell (LT-HSC) population and in all maturing stages of the haematopoietic hierarchy [6, 29]. This persistence of a MPN stem cell population can explain relapse of MF post allogeneic transplant. LT-HSC cells within the marrow are critical for initiation and maintaining the disease process [30]. Yet, these *JAK2* V617F LT-HSC population appear to exhibit reduced self-renewal and are skewed towards expansion of the progenitor pool instead [31]. In murine models of *JAK2* V617F MPN this defective self-renewal of LT-HSCs is rescued by acquisition of a concurrent *TET2* mutation [32]. Given the heterogeneity in normal haematopoietic stem cells, the original bias of the stem cell acquiring the driver mutation may impact on disease progression and phenotype [33].

**Fig. 1:**
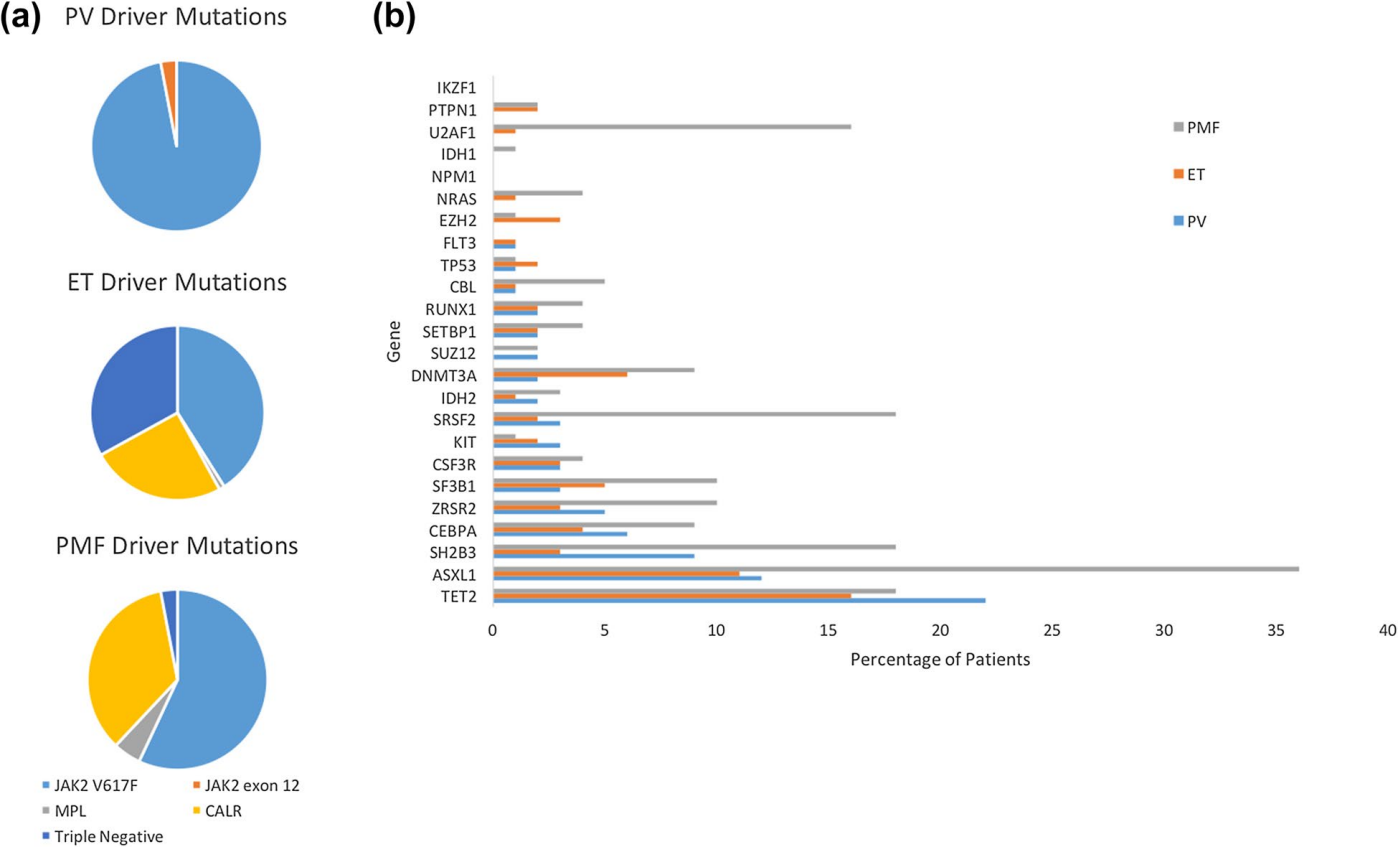
**a** This demonstrates the relative proportions of driver gene mutation observed in each MPN phenotype. **b** This demonstrates the proportion of patients with each MPN phenotype with a mutations in a panel of genes commonly mutated in myeloid malignancy. Frequency of mutations presented is based on data acquired in by Tefferi et al. [34, 35]

A range of genes are repeatedly found to be mutated in MPN and across the spectrum of myeloid disorders. These co-operating oncogenic mutations found alongside the driver mutations include genes involved in cell signalling pathways (*LNK*, *CBL*, *NRAS* and *NF1*), epigenetic regulation (*ASXL1*, *EZH2*, *TET2*, *DNMT3A*, *IDH1* and *IDH2*), transcriptional regulation (*TP53*, *RUNX1)* and mRNA processing (*SF3B1*, *SRSF2*, *U2AF1*, *ZRSR2*). Using targeted next generation sequencing (NGS) of 104 cancer‐related genes on 197 MPN patients, approximately 10% of patients had no mutation detectable in any of the genes analyzed and 54% had mutations only in *JAK2 V617F* or *CALR.* The remaining 36% had additional mutations detected, other than *JAK2 V617F* or *CALR*. Most of these were mutations affecting genes implicated in epigenetic regulation [27]. Figure 1 shows a chart representing the relative proportions of driver mutations and additional mutations detectable in PV, ET and PMF [34, 35]. These genes may occur concurrently within clones containing the driver mutation, in sub-clones without the driver mutation and at different levels of the haematopoietic cell hierarchy and impact on phenotype and prognosis [27].

Understanding the impact of this genomic complexity and clonal evolution on the MPN phenotype, pathogenesis and response to JAKi is critical to improving our therapeutic approach.

### *JAK2* V617F, *CALR* and *MPL* exon 10

At the driver gene level for instance, the presence of the *JAK2* V617F mutation results in a higher thrombotic risk in ET and PMF compared to *JAK2* negative cases [36]. Gene expression patterns consistent with JAK/STAT activation are also more evident patients with allele amplification [8]. Homozygosity or higher *JAK2* V617F allele burden has been suggested to emphasise the PV phenotype with a higher haematocrit and greater rate of fibrotic transformation observed [37]. Yet, homozygosity for *JAK2* V617F is a commonly occurring event in MPN and can be seen in patients with a PV, ET or PMF phenotype whilst the same is true for heterozygotes. *JAK2* V617F is also detectable in patients without overt haematological malignancy phenotypes, the so termed “clonal haematopoiesis of indeterminate potential” (CHIP). Indeed, estimates of *JAK2* mutation prevalence are higher in CHIP than in MPN [38, 39]. This is suggestive that additional changes are required for a MPN phenotype to develop. There is inconclusive evidence that the allele burden of *JAK2* V617F impacts on thrombosis risk with conflicting evidence of effect in a number of studies [40]. A unique cohort of patients with splanchnic vein thrombosis and underlying *JAK2* V617F positive mutant clones tend towards a lower allele burden [41]. In CHIP the presence of mutated *JAK2* results in a significantly increased coronary artery disease risk despite the absence of the MPN blood phenotype [42]. It is therefore clear that the presence of mutant *JAK2* V617F plays a critical role in increasing thrombotic risk. This thrombosis risk appears to be mediated in part by viscosity from increased haematocrit, in part by increased binding to endothelial laminin as a result of an *JAK2* V617F driven activation of Lu/BCAM [43] and from increased neutrophil extracellular trap formation [44]. It is not clearly related to an increasing burden of *JAK2* V617F clonal cells present.

RUX has demonstrated a limited impact on the allele burden of mutant *JAK2* in the clinical trials to date in PV and PMF but not ET. COMFORT-II demonstrated a reduction in allele burden of greater than 20% in 38.3% of RUX treated MF patients at 168 weeks [14]. In PV, post hoc analysis of the RESPONSE trial at 208 weeks has shown a clear and progressive reduction in *JAK2* V617F allele burden with continued treatment with average reductions of around one-third achieved in the RUX randomised arm [45]. However, correlations with haematological parameters of disease (haematocrit level, leucocyte count, platelet count) were not observed [45]. This therefore provides a challenge to the use of *JAK2* allele burden reduction as a relevant biomarker of treatment success. One small study has suggested patients starting with a higher allele burden may benefit the most from RUX therapy in MF with a study of 69 patients showing a higher probability of spleen response if allele burden was greater than 50% at entry [46].

The presence of the *CALR* driver mutation meanwhile appears to offer some protection. Despite the observed higher platelet counts in *CALR* positive ET, there is a lower thrombosis risk [47]. Indeed, the use of anti-platelet agents in low risk *CALR* positive ET may cause harm rather than provide a benefit [48]. Overall survival is also comparatively higher in *CALR* positive MF [47]. The rate of leukaemic transformation in *CALR* patients was similar to *JAK2* V617F patients in a meta-analysis of twelve studies in PMF [49]. The presence of type 1 *CALR* mutations was prognostically favourable for overall survival with regards to type 2 *CALR* mutations in PMF. However, multivariate analysis incorporating additional co-mutations and prognostic scoring systems did not retain this association [50]. Patients with triple negative mutation status in PMF have a faster rate of disease progression and leukaemic transformation and worse overall survival than any of the driver mutations [47].

The effect of JAK inhibition with RUX in MF does not appear to be affected by the underlying driver mutation. COMFORT-I showed no difference in clinical effect between *JAK2* V617F positive or negative patients [10]. A further exploratory analysis of the COMFORT-II study showed no difference in the response of *CALR* mutant patients in comparison to the cohort as a whole [51]. *CALR* mutant mice develop an ET phenotype that is ameliorated by RUX [52]. Likewise, murine models of *JAK2* V617F positive MPN show response in spleen weight and haematological parameters to the administration of RUX [53]. Another JAK2 inhibitor INCB16562 showed efficacy in a murine model of *MPL* W515L induced thrombocytosis and myelofibrosis [54]. This is logical given the activation of the JAK/STAT pathway in all mutant backgrounds.

The MPN HSC population appear to escape JAKi with limited impact on quantitative reduction in the MPN HSC population demonstrated even in in vitro treatment. This is part of the key to explaining why molecular remissions are rarely achieved and why MF patients often rebound quickly after discontinuation of these drugs [55].

### Co-operating Mutations and Clonal Evolution

Recurrently mutated co-operating oncogenes have key roles in cell signalling pathways, epigenetic regulation, transcription regulation and mRNA processing. In PMF, the presence of the mutations in the genes *ASXL1*, *EZH2*, *SRSF2* and have were observed to independently predict shortened survival in a European cohort of 483 patients and validated in an American cohort of 396 patients. In both cases *ASXL1* retained this prognostic relevance when prognostic scoring systems were accounted for. In both cohorts.

*IDH1/2* and *SRSF2* mutations were associated with leukaemic transformation whilst *ASXL1* was also associated in the European cohort [56]. *TP53* mutations were not analysed in that study, however somatic mutations with loss of heterozygosity in *TP53* was strongly associated with progression to a leukaemic blast phase in a cohort of 197 patients analysed with next generation sequencing. Two or more somatic mutations was associated with the same negative prognosis [27]. *JAK2* V617F cooperates with loss of *TP53* in a murine model to induce blast phase disease. A larger cohort of 797 patients across Europe and North America demonstrated a statistically significant negative impact on overall survival and leukaemia free survival when two or more of *ASXL1*, *EZH2*, *SRSF2* and *IDH1/2* were mutated [57]. There is also evidence of reduced progression free survival following allogeneic haematopoietic stem cell transplantation for MF patients carrying *ASXL1* and *IDH1/2* at time of transplantation [58].

Some genes are strongly associated with particular MPN phenotypes, for example, mutated *ASXL1* is documented in around 38% of PMF patients and a much lower percentage of other MPNs [59]. Genes involved in splicing are more commonly mutated in PMF [26, 60]. Others, for example, *IKZF1* are almost exclusively identified in blast phase disease [61]. The majority of PV and ET patients possess only one identified mutation in a driver gene whilst a much higher number of PMF patients have multiple somatic mutations. This is suggestive that acquisition of particular mutations distorts the equilibrium in favor of a particular phenotype. It has been suggested that the acquisition of co-operating mutations promotes a shift towards dysplasia from proliferation and therefore PMF could be considered as an additional MPN/MDS overlap syndrome [26].

*TET2* is commonly mutated in the MPNs and myeloid malignancy in general but shows no particular phenotypic bias. It was suggested in one study to confer a poor risk prognosis for overall survival and blast crisis transformation [27]. It is also frequently detectable in CHIP [62]. Timing of acquisition of this mutation appears to play a key role in the resulting disease phenotype. *TET2* first cells have a proliferation advantage at the haematopoietic stem cell (HSC) level but do not result in excess production of mature megakaryocytes or erythroid cells until acquisition of a concurrent *JAK2* V617F mutation. Cells acquiring the *JAK2* V617F mutation first do not expand at the HSC level but can produce excess numbers of erythroid and megakaryocyte cells through proliferation of progenitors. Expansion is enhanced by subsequent acquisition of the *TET2* mutation. These *JAK2* V617F first cells appear to favour a polycythaemia phenotype, with an increased risk of thrombosis in the patient group whilst the transcriptional profile of the cells was significantly altered dependent on the first acquired mutation [63]. In contrast “*TET2* first” cells promote an ET phenotype. Greater than 70% of MF patients with *EZH2* mutations also harbored *ASXL1* mutations in one study. These two events were observed in the earliest multipotent HSC population. Acquisition of these mutations in this context appears to have preceded the acquisition of *JAK2, CALR* or *MPL* mutations in many cases [59]. The order of genomic event acquisition therefore appears to be a key feature in determining the subgroup of MPN, with each event potentially providing different selective advantages at varying stages of the hematopoietic hierarchy and resulting in a different biological outcome.

This resultant changing genomic landscape and acquisition of new mutations allows for the evolution of the clonal cell populations over time. In the chronic phase of disease this appears to be a very slow process with only two new mutations detected during follow-up equivalent to 133 patient years in one study across MPN [27]. Dominance can then established by one clone whilst others may disappear over time. Co-operating mutations may occur alongside the driver mutations or in separate clones. Recent work has demonstrated the accumulation of low burden of *TP53* mutant clones associated particularly with ageing over the course of chronic MPN. This has demonstrated that multiple variants of these *TP53* may exist concurrently [64]. The clonal landscape may also vary at different points of the haematopoietic hierarchy [63]. The most devastating result of clonal evolution is the selection and expansion of a clone resulting in blast phase of disease. The leukaemic clone may not even contain the original driver mutation and patients with *TP53* mutations may develop leukaemic clones expressing wild type *TP53* [64, 65]. The higher prevalence of mutations detectable in blast phase disease is suggestive of a higher rate of mutation acquisition similar to that seen in blast phase CML [66].

There is also interest in the role of germline genomic susceptibility factors in MPN. There are cases of familial MPN characterised by a number of rare germline mutations including *RBBP6*. The JAK2 46/1 combination of haplotypes is also associated with both *JAK2* V617F positive and *MPL* positive MPN and is suggested to impact of a clonal advantage for any cells acquiring these mutations [67]. An in depth review of these germline factors is however beyond the scope of this article as the impact on the effectiveness of RUX has not been clearly evaluated.

We are moving towards a time when molecular markers will help to provide individualized prognostication and reveal a spectrum of phenotype, thrombosis and transformation risk [68]. It is in this spectrum of disease that the role of JAKi and RUX in particular will need to be defined. Molecular data from the initial RUX clinical trials is rather limited in this respect as is shown in Table 1. Analysis of the COMFORT-II study in PMF shows that when patients were grouped into a high molecular risk group according to the presence of mutation(s) in any one of *ASXL1*, *EZH2*, *SRSF2*, and *IDH1*–*2 or* low molecular risk group as had previously been validated [56], this did not affect the likelihood of obtaining a > 35% spleen volume reduction or symptomatic improvement [13]. However, subsequent analysis of Phase1/2 studies using RUX in MF including post PV or post ET-MF suggested that the presence of three or more mutations was associated with a worse spleen response and shorter time to treatment discontinuation [69]. Another recent small study suggested that the presence of *ASXL1* or *EZH2* was independently associated with an inferior time to treatment failure on multivariate analysis in a study of 100 MF patients treated with RUX or momelotinib [70]. Therefore, the underlying genomic landscape may affect RUX efficacy. In PV, the 208 week evaluation of data from the RESPONSE-II trial reported on rates of complete (CMR) and partial molecular response (PMR). In the case of patients originally randomised to RUX, CMR and PMR were possible in patients with *ASXL1* and *TET2* mutations identified. The prognostic implications of these mutations is less clear in PV but again this suggests that in particular individuals, that at least in certain individual circumstances the effectiveness of RUX is not impacted by the presence of these mutations [45]. Of course, the definition of CMR or PMR was based on *JAK2* V617F mutation burden and whether patients with CMR or PMR obtained simultaneous reductions in *ASXL1* or *TET2* allele burdens was not reported.

Direct therapeutic targeting of *IDH2* using small molecule inhibition alongside RUX has shown superior efficacy to monotherapy in *JAK2* V617F and *IDH2* mutant murine MPN models and synergistic effects in dual mutated primary MPN cells [71]. This highlights the potential for direct targeting of mutant cells using personalised therapy guided by the mutational landscape for select patients.

In blast phase, response to conventional systemic chemotherapy for acute myeloid leukaemia (AML) has a limited efficacy [72]. Median survival is less than 6 months which can be improved in patients undergoing allogeneic stem cell transplantation [73]. However, many patients are not fit for this intensive treatment approach. RUX monotherapy was effective at improving survival in a murine model transplanted with *TP53* knockout/*JAK2* V617F positive leukaemic cells but was insufficient to induce remissions and was inferior to combination therapy with a histone deacetylase inhibitor (HDACi) or a Heat shock protein 90 inhibitor [74]. Clinical data is sparse but small numbers of patients have shown improved responses in combination with intensive chemotherapy induction and alongside HDACi [75–77]. In MF, patients with an excess of blasts between 5 and 9% in bone marrow or peripheral blood demonstrated an improved response to RUX which was not seen for those with an accelerated phase defined by 10–19% blasts [78]. These results suggest that targeted JAKi with RUX may have a role in the treatment of blast phase disease but optimizing synergy with additional agents is likely to be the key to improving therapeutic efficacy in this scenario.

When it comes to clonal evolution understanding the impact of RUX is going to be critical. Analysis of phase 1/2 trials of RUX in MF allowed analysis of molecular profile from 62 patients at baseline and at discontinuation of RUX therapy. Just over one-third of these patients acquired further mutations whilst on therapy. These included *ASXL1, TET2, EZH2* and *TP53* most frequently. This clonal evolution was associated with shorter survival following RUX discontinuation. Half of patients with molecular data available that transformed to AML did so on the background of clonal evolution during RUX therapy [79]. There have been reports of an usually high occurrence of extramedullary leukaemia whilst on RUX [80]. However this does not appear to be an observed phenomenon in the clinical trial setting or repeatedly reported and therefore may not be significant. Monotherapy in malignancy is frequently associated with clonal escape and it is not clear whether any selective pressure driving this is applied by RUX. Recent work has suggested that around 15% of MF patients have demonstrable Immunoglobulin gene rearrangements (IgR) in bone marrow indicative of a B Cell clonal population. They further observed an incidence of aggressive B-Cell lymphoma in 5.8% of RUX treated patients compared to 0.36% of patients not exposed to this agent. All of these patients had prior detectable IgR. This is suggestive of an ability of RUX to select for a lymphoid clone possibly through immunosuppressive effect [81]. Whether the same may be applicable for myeloid clones is less clear but must be carefully evaluated as clinical trials of RUX as a front line agent in PV are developed.

### Cell signaling pathways

**Fig. 2:**
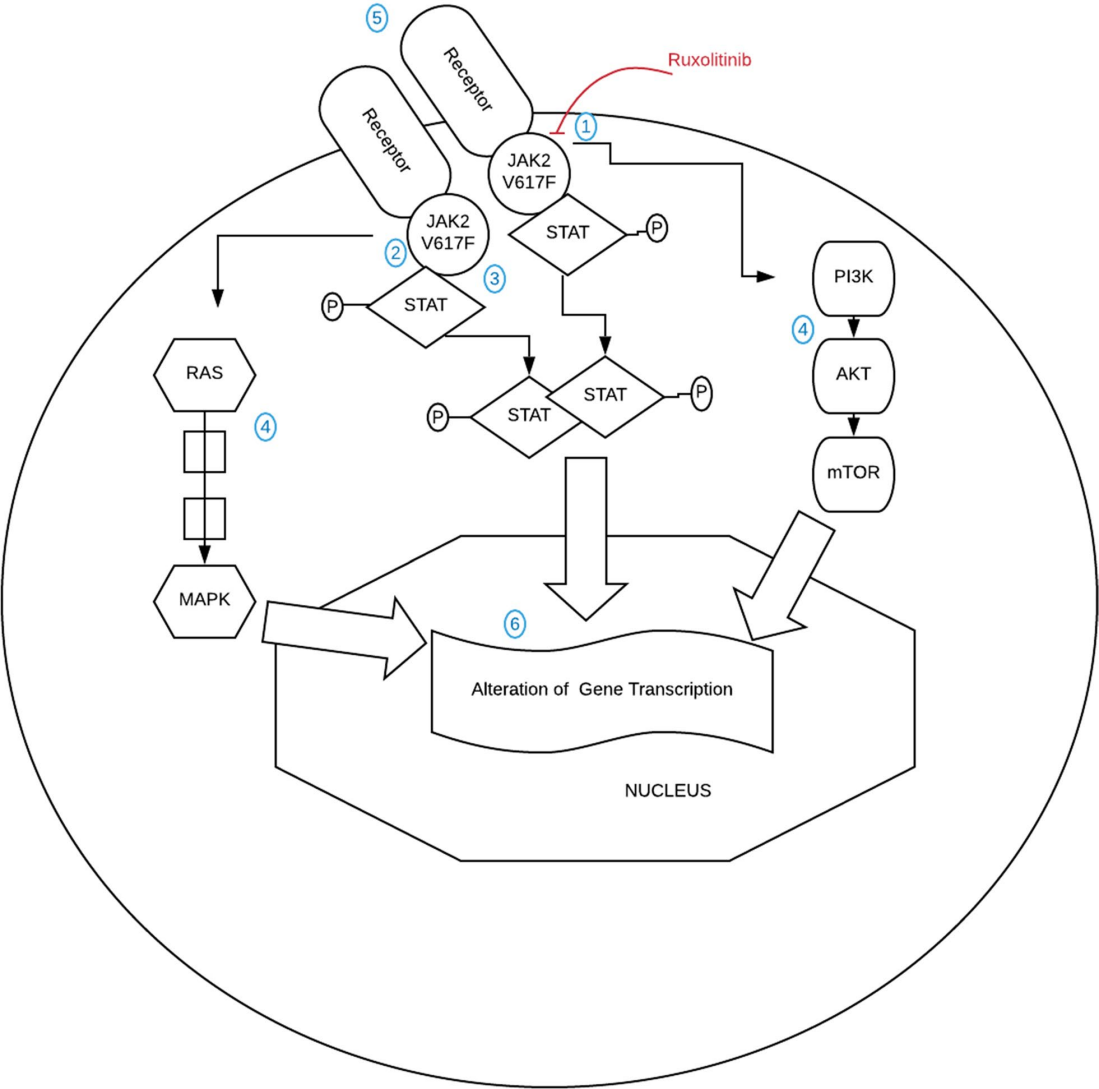
This schematic demonstrates the potential mechanisms of escape from JAK2 inhibition by ruxolitinib. (1) Ineffective JAK inhibition (2) Acquired tyrosine kinase domain mutation (3) Heterodimerization of JAK2 with JAK1 or TYK2 (4) Alternative signaling cascade activation (5) External cytokine effects (6) Epigentic mechanisms of transcriptional regulation

The persistence of MPN clones despite JAKi shows that the cells are able to escape the inhibition blockade and survive. Figure 2 demonstrates the signaling cascade activated by constitutive JAK2 activation highlighting a number of potential mechanisms of escape that have been demonstrated.

In many malignancies, resistance to tyrosine kinase inhibitor (TKI) therapy occurs on the basis of an acquired mutation with a key drug target. This is demonstrated in chronic myeloid leukaemia (CML) when acquired resistance to TKIs is often the result of a new mutation within the *BCR*-*ABL1* fusion gene. Saturation mutagenesis studies in murine cell line with RUX have demonstrated the emergence of second site mutations within *JAK2*. These mutations conferred resistance to JAKi by a number of agents including RUX [82, 83]. However, it is not clear that this is a relevant phenomenon which is occurring in MPN patients [84] and persisting clones demonstrate the absence of second site mutations in the presence of JAKi [85]. This suggests that the dosing of RUX is insufficient to exert a selective mutagenesis pressure in patients. Genetic deletion of *JAK2* in a murine model of *MPL* mutated MPN was more effective than JAKi in ameliorating the disease state demonstrating the potential benefit of improved JAKi. When persisting *JAK2* V617F cell lines in RUX were examined it was evident that there was reactivation of JAK/STAT signaling. Dimerisation is a critical aspect of JAK2 activation and in the presence of RUX this can occur as a heterodimer between JAK2 and JAK1 or TYK2 resulting in a reactivation of JAK/STAT signaling and resistance to JAKi [85]. This is a functional mechanism of resistance and appears to be reversible on withdrawal of RUX with cells re-sensitizing over a period of weeks. It is interesting to note patients may demonstrate similar responses to RUX re-challenge following withdrawal for loss of response [86].

Another possible mechanism of resistance to RUX is the recruitment of alternative cell signaling pathways continuing to drive the disease phenotype. Constitutive activation of JAK/STAT signaling in *JAK2* V617F positive cells is accompanied by activation of the STAT independent PI3K or MAPK pathways [4]. CALR mutations also activate MAPK signaling [87], however, there does appear to be a differential expression profile in MAPK and PI3K pathways in *CALR* mutant ET compared to *JAK2* V617F mutant ET or PV [88]. These role of these pathways in the pathogenesis of MPN and the resistance to JAKi is beginning to be elucidated. The PI3K/mTOR pathway is critical for the phosphorylation of serine residues on STAT5b. These phosphorylations on serine-193 and serine-731 residues were not affected by exposure to RUX but were reduced by treatment with PI3K or mTOR inhibitors in *JAK2* V617 positive cell lines. Combinations of RUX with PI3K and mTOR inhibitors was more efficacious in cell lines, primary patient cells and knock in mouse models of *JAK2* V617F positive MPN [89]. Indeed, an mTOR inhibitor everolimus has been trialed in MF showing some efficacy in symptom burden control and spleen volume reduction [90]. Trials to understand the efficacy of RUX in combination with these inhibitors are yet to report on efficacy in real world patient samples.

### Epigenetic dysregulation beyond genomic mutations and cell signaling pathways

Histone modification is a key mechanism of epigenetic regulation with n-terminus lysine residues undergoing post translational modifications including acetylation, methylation and phosphorylation which can enhance or repress gene transcription. We have already discussed the occurrence of mutations in genes controlling histone modification including *ASXL1* and *EZH2* which are frequently observed in MPN. In murine studies, differential distribution of acetylated H3K27 between progenitors in *MPL* W515 mice with an MF phenotype and controls was observed. Using a chromatin immunoprecipitation assay the active loci marked by acetylated H3K27 were significantly associated with Tumor necrosis factor(TNF)/nuclear factor KB(NF-KB) signaling pathways highlighting a key role of inflammation which will be discussed below [91]. Some mechanisms of histone modification have been proposed. The mutant JAK2 V617 protein is able to locate to the nucleus of the cell. In doing so, it is able to exert effects through phosphorylation of Histone H3 and the arginine methyltransferase PRMT5 resulting in histone modification and change gene transcription [92, 93]. Over-expression of the transcription factor “nuclear factor erythroid 2” (NFE2) occurs in the majority of MPN patients. This over-expression results in significantly elevated levels of the histone demethylase JMJD1C and resultant global reduction of H3K9me1 and H3K9me2 levels [94]. There is limited published work on the effect of JAKi on histone modifications. We have demonstrated modifications to histone methylation at lysine 36 on Histone H3 in response to RUX therapy and shown that patients with high levels of di and tri methylation at lysine 4 may have sub-optimal responses to RUX therapy [95]. A number of histone deacetylase inhibitors have been investigated as therapy in MPN. Only Panobinostat, a pan-deacetylase inhibitor which enhances acetylation at H3 and H4 histones, has been investigated in combination with RUX in a phase 2 study. They observed greater reductions in splenomegaly than were expected for RUX therapy only [96]. Lysine specific demethylase 1 (LSD1) modifies methylation at histone H3 by removal of methyl groups. Inhibition of LSD1 by small molecule is beneficial in murine models of MPN and synergistic with RUX in ameliorating the MPN process in mice. JAKi alone may be insufficient to overcome effects of prior histone modification. The role of combining epigenetic manipulation and JAKi needs explored in clinical trials [97].

DNA methylation (DNAm) is a further mechanism of transcriptional regulation [98]. Changes in DNA methylation are observed in normal ageing and pathology. DNA methyltransferase enzymes (DNMT) act to methylate cytosine residues at CpG sites silencing transcription. Genes involved in this process that are commonly mutated in MPN include *DNMT3A*, *TET2* and *IDH1/2.* Samples from MPN patients show aberrant DNA methylation patterns in comparison to controls and this changes again during transformation to blast phase of disease [99]. Using an ageing signature based on DNAm patterns in key genes [100], we have demonstrated that PV patients have a DNAm age which is older than their chronological age whilst ET patients tended to have a younger DNAm age. Interestingly, following therapeutic intervention with RUX, the DNAm age, in both groups, moved closer to their actual age [95]. Whether this difference in DNAm pattern is reflective of a direct influence on the MPN phenotype or reflects the actions of other cellular processes is unclear but does however demonstrate another feature of epigenetic dysregulation in these pathologies.

DNAm and histone modification represent pre-transcriptional mechanisms of control. Increases or decreases in gene transcription levels may be further modified by post-transcriptional or post-translational modification which may impact on the resulting proteome and ultimately the impact on the cell processes. Quantitative mass spectrometry has shown differences in the make-up of a small proportion of the proteome across different MPN phenotypes and mutational backgrounds. Proteins in the RAS GTPase and oxidative stress pathways were identified as differentially expressed. CALR was noted to be over-expressed in *JAK2* V617F positive backgrounds in comparison to wild type backgrounds suggesting that the presence of mutant JAK2 may be able to impact on wild type CALR expression which may impact on cellular signalling impacting on phenotype [101]. This CALR overexpression could be ameliorated by JAKi in murine cell line culture [101]. To our knowledge, there has been no data on the effect of RUX or JAKi on the proteome of treated patients.

### DNA damage

Given the propensity of MPN cells to acquire multiple genomic aberrations, a number of efforts to understand the role of DNA damage accumulation and repair mechanisms have been undertaken. A number of mechanisms linking genome instability have been proposed. Activation of *JAK2* V617 has been shown to stimulate increased homologous recombination activity and can result in chromosome centrosome abnormalities and an increased rate of mutagenesis in vitro [102]. This may promote a loss of heterozygosity of *JAK2*. PI3K dependent signalling results in replication fork stalling and activation of the intra- S phase cell cycle checkpoint. The latter effect was only observed in PV erythroblasts and not in ET erythroblasts [103]. Reactive oxygen species appear to play a key role in the excess accumulation of double strand DNA breaks (DSBs) that have been observed in MPN cells [104, 105]. This was accompanied by avoidance of usual apoptotic mechanisms in response to DSBs and may help to explain the accumulation of additional mutations evident in many MPN cells [104]. Downregulation of NHE1/BCL-xl pro-apoptotic pathways via PI3K mediated inactivation of FOXO3A allows inappropriate cell survival despite DNA damage accumulation. This mechanism appears to be differentially present in stem cells in comparison to progenitors [106]. Anti-oxidative therapies were effective in reducing the number of DSBs acquired in *JAK2 V617F* positive mouse model and in reducing the progression of the MPN phenotype demonstrating the importance of this genomic instability in pathogenesis [104]. *JAK2 V617F* expression can negatively regulate p53 via degradation by increased levels of MDM2 thus impacting on the critical role p53 plays in the DNA damage response [107]. Downregulation of DNA repair pathways mediated by *BRCA1* and *ATM* has been demonstrated in *CALR* mutants by gene expression profiling [88]. RUX has been demonstrated to inhibit BRCA-mediated homologous recombination and DNA—dependent protein kinase-mediated non homologous end joining. This leads to an accumulation of DSBs which lead to synthetic lethality in cell line cultures, primary MPN samples in vitro and xenograft models when combined with poly-ADP-ribose polymerase (PARP) inhibitors [105]. Given that RUX is poor at inducing apoptotic pathways [108], this reduction in the DNA repair mechanisms in cells that are prone to increased DNA damage may potentially allow for clonal selection in monotherapy highlighting the critical need for vigilance for clonal evolution in front line RUX trials.

### Inflammation

Neoplastic disease has been linked to inflammation in many ways, including, as a driver of malignant change, sustaining the disease or resulting from the neoplastic cells. Levels of circulating cytokines are higher in MPN patients. A number of studies have demonstrated prognostic value for IL-8, IL-2R, IL-12, IL-15 and high sensitivity C reactive protein (CRP) levels in MF [109, 110]. Both mature and progenitor cells demonstrate an aberrant cytokine production pattern with differences evident between the two [111]. Cytokine profiles are also different between the MPN phenotypes with higher levels seen during transformation of disease to secondary MF or AML [112]. In the general population, inflammation is linked to thrombosis and increasing high sensitivity CRP levels correlate with thrombosis risk in ET and PV [113]. Constitutional symptoms in MPN are similar to those observed in other inflammatory disorders. The influence of pro-inflammatory cytokines on bone marrow fibrosis has been demonstrated therefore showing a direct impact on the MPN phenotype [114]. These pro-inflammatory cytokines result from both mutant haematopoietic MPN clones and non–mutant haematopoietic cells as a direct result of JAK/STAT signalling driven by cytokine influences [111]. Therefore a self -reinforcing cycle of inflammation is created. Recent investigation has shown that changes in the chromatin landscape by altered methylation and acetylation patterns at histone H3 lysine 4 and lysine 27, respectively links with increased expression of NF-KB signalling pathways driving associated inflammation [91].

RUX has demonstrated good efficacy in an anti-inflammatory role. It is efficacious in graft versus host disease and is under investigation in other immune mediated conditions [115]. A reduction in cytokine levels in MPN during RUX therapy is observed and the constitutional symptom burden is generally reduced. The reduction is spleen burden resulting from extramedullary haematopoietic activity may also reflect a reduction in inflammation. This anti-inflammatory effect can be augmented through the use of BET inhibitors to disrupt the epigenetic enhancement of NF-KB signalling. In murine model of *JAK2* V617F MF, the combination of RUX and BET inhibition resulted in significant reversal of bone marrow fibrosis and a reduction of disease burden [91]. Therefore targeted therapeutic manipulation of the pro-inflammatory pathways appears to be an efficacious strategy which is worth further investigation in patients.

### Future directions of therapy

The ground breaking efficacy of TKI monotherapy in CML has not been replicated in MPN through the use of targeted JAKi with RUX. Yet, the advances in our knowledge have revealed complexities in genetic landscape, epigenetic dysregulation, signaling cascades, DNA damage response and inflammatory pathways. Each of these abnormalities underlies the pathogenesis and impacts on the effectiveness of JAKi in these disorders. Augmentation of JAKi through concurrent therapeutic manipulation of alternative pathways is a key focus of current research. Clinical trials are underway to evaluate the benefit of RUX alongside epigenetic modifiers, immunomodulatory drugs, small molecular inhibitors of PI3 K/AKT/mTOR and Interferon [116]. Understanding how these combinations affect the burden of disease, level of bone marrow fibrosis and risk of progression is critical to establishing efficacy beyond the parameters demonstrated already by JAKi. As accurate personalized risk profiles become achievable based on genomic data, understanding how RUX fits the treatment for each of these individuals will be important. Development of JAKi with increased activity and/or allosteric inhibition alongside agents with specificity for mutant JAK2 may be significant in the coming years [117, 118]. Finally, effective eradication of the MPN stem cell niche will be required to bring about disease cure.

## Conclusion

The advances in genomic and epigenetics over the last number of years have helped to reveal significant amounts of information regarding the pathogenesis of MPN. Rather than distinct disease entities, there is a complex evolving spectrum of pathology with common features and key differences. The role of constitutive activation of the JAK/STAT pathway is common across the spectrum whilst the role of co-operating mutations, epigenetic dysregulation, clonal evolution, responses to DNA damage, activation of cell signaling pathways and inflammatory activation varies resulting in differences in the observed MPN phenotype, progression of the disease and risk of thrombotic complications. This is allowing a move away from simple grouping by phenotype in the clinic towards classification by increasingly towards a biological underpinnings of the phenotype. JAKi has demonstrated good efficacy in symptom relief but more limited impact on disease modification and there are concerns regarding the impact on clonal landscape that need careful evaluation. Understanding how JAKi affects and is affected by each of the key features of pathogenesis above is key to understanding how best to augment this therapy and establish an optimal therapeutic approach to this complex disease state.

## Abbreviations

WHO: World Health Organisation
PV: polycythaemia vera
ET: essential thrombocythemia
PMF: primary myelofibrosis
MPN: myeloproliferative neoplasms
MF: myelofibrosis
EPOR: erythropoietin receptor
MPL: thrombopoietin receptor
GCSF: granulocyte-colony stimulating factor receptors
JAKi: JAK inhibition
RUX: ruxolitinib
HU: hydroxycarbamide
BAT: best available therapy
OS: overall survival
LT-HSC: long term haematopoietic stem cell
NGS: next generation sequencing
CHIP: clonal haematopoiesis of indeterminate potential
mRNA: messenger RNA
MDS: myelodysplastic syndrome
HSC: haematopoietic stem cell
CMR: complete molecular response
PMR: partial molecular response
AML: acute myeloid leukaemia
HDACi: histone deacetylase inhibitor
IgR: immunoglobulin rearrangement
CML: chronic myeloid leukaemia
DNAm: DNA methylation
DNMT: DNA methyltransferase enzymes
DSB: double strand DNA breaks
CRP: C reactive protein
